# Tuning the Oxidative Activity of Single Atom Catalysts by Carbon Doping in Hexagonal Boron Nitride Supports

**DOI:** 10.3390/nano16010061

**Published:** 2025-12-31

**Authors:** Jie Zhang, Yingguang Zhou, Naixia Lv

**Affiliations:** College of Biology and Chemistry, Minzu Normal University of Xingyi, Xingyi 562400, China; z748586074@163.com (J.Z.); zhou770927@163.com (Y.Z.)

**Keywords:** single-atom catalysts, O_2_ adsorption, BN support, carbon-doping

## Abstract

Single-atom catalysts (SACs) have gained significant attention due to their exceptional metal atom utilization efficiency and high catalytic activity. Using DFT calculations, single-atom metals (M = Ag, Au) on defective and carbon-doped h-BN supports (M@BN and M@nC-BN) are systematically investigated to elucidate the effects of C-doping concentration and configuration on their structural stability, and to explore their potential application in O_2_ activation. The results indicate the singlet O_2_ adsorbed configuration is more effective in activating the O–O bond than the triplet one. Ag@4C-BN and Au@6C-BN exhibit good stability comparable to their undoped counterparts. Compared to M@BN, the M@nC-BN surfaces, particularly M@4C-BN, exhibit significantly enhanced adsorption of singlet O_2_, accompanied by the most notable O–O bond elongation, indicating its superior capability for O_2_ activation. DOS and frontier orbital analysis reveals that C-doping upshifts the HOMO energy level of M@4C-BN, endowing the catalyst with a stronger electron-donating ability to O_2_ 2π* and leading to efficient activation. This study provides a theoretical basis for the rational design and optimization of BN-based single-atom catalysts.

## 1. Introduction

Single-atom catalysts (SACs), characterized by atomically dispersed metal active sites on support surfaces, achieve high metal atom utilization efficiency [1,2,3]. This makes them an ideal platform for developing cost-effective catalysts, particularly those based on precious metals like gold, silver, platinum, and palladium et al. Consequently, SACs have emerged as a prominent class of high-efficiency catalysts in recent years [4,5]. The catalytic performance of SACs combines the advantages of both homogeneous and heterogeneous catalysts. Their uniform active sites often confer high selectivity for specific reactions, rivaling the performance of homogeneous catalysts, while simultaneously offering the ease of separation and recovery inherent to heterogeneous catalysts. These attributes make SACs promising for broad applications in electrocatalytic reduction, oxidation, hydrogen production, and related fields [6,7,8,9,10,11,12,13,14,15,16,17]. However, isolated metal atoms, due to their high surface energy, tend to agglomerate into nanoparticles during synthesis and catalytic reactions. Studies indicate that constructing strong electronic or covalent interactions between metal atoms and the support via appropriate synthesis strategies is key to stabilizing single atoms and preventing their migration and agglomeration [18,19,20].

Hexagonal boron nitride (h-BN), composed of alternating B and N atoms arranged in a two-dimensional layered honeycomb structure, exhibits key properties such as a large specific surface area, oxidation resistance, thermal stability, and chemical inertness [21,22,23]. These characteristics make h-BN an ideal support material for anchoring active sites like polyoxometalates (POMs), metal oxides, metal nanoparticles (NPs), and single metal atoms, enabling the construction of highly active composite catalytic materials for various reactions [24,25,26,27]. Notably, vacancy defects in h-BN layers can be created via electron beam irradiation [28,29]. These defective structures can effectively anchor single transition metal atoms. Furthermore, theoretical and experimental studies confirm that boron vacancies (V_B_) form more readily than nitrogen vacancies (V_N_) in h-BN [30,31]. Theoretical calculations suggest that h-BN sheets containing V_B_ are highly promising supports for single transition metal atoms [32,33,34,35,36]. h-BN is a typical electrical insulator with a wide bandgap (~6 eV) [37]. Incorporating carbon atoms into the h-BN lattice significantly alters its electronic structure, reducing the bandgap to a tunable range of 0–5.5 eV. Therefore, C doping serves as an effective strategy for modulating the electronic structure of h-BN [37,38,39]. Previous studies have shown that C-doped h-BN as a support exhibits excellent catalytic performance in adsorptive desulfurization, CO_2_ capture, and the nitrogen reduction reaction [40,41,42].

In catalytic oxidation field, O_2_ is considered an ideal oxidant due to its safety and low cost [43]. However, the high chemical stability of O_2_ makes its adsorption and activation a critical initial step in many such reactions [34,44,45]. Moderate O_2_ adsorption strength on the catalyst surface and efficient activation to generate reactive oxygen species (e.g., O_2_^−^, O_2_^2−^) are essential for driving various oxidation processes [44,46]. This study will design single-atom catalysts (SACs) with metal atoms M (M = Ag, Au) anchored on hexagonal boron nitride (h-BN) and carbon-doped BNC monolayers, aiming to investigate the effects of carbon doping concentration and configuration on the stability of these SACs and their catalytic reactivity toward oxygen activation. The findings are expected to provide theoretical guidance for the design of SACs with good stability and superior catalytic activity.

## 2. Computational Methods and Models

### 2.1. Computational Methods

The calculations in this study were performed using a hybrid density functional method containing dispersion correction (B3LYP-D3). For the Au and Ag metal atoms, the Stuttgart/Dresden effective core potential (SDD) basis set were employed. This basis set adopts an effective core potential to replace the inner-shell electrons of heavy metals, while retaining valence electrons for detailed calculation; more importantly, it incorporates relativistic effects [47]. For non-metal atoms (B, N, C, H, and O), the all-electron polarized triple-zeta basis set 6-311G(d, p) was used. During the geometry optimization process, all atomic configurations were fully relaxed with the adoption of stringent convergence criteria. Specifically, the convergence cutoff for energy computations was configured to 1.0 × 10^−8^ atomic units (a.u.), while for the geometry relaxation process, the maximum force tolerance and maximum displacement limit were set to 0.00045 a.u. and 0.0018 a.u., respectively. Previous research has confirmed this computational method is suitable for simulating metal interactions with h-BN systems [48]. To further verify the rationality of the optimized structures, frequency calculations were performed. Structures without imaginary frequencies were confirmed as local minima, while those with exactly one imaginary frequency were identified as transition states. All theoretical computations were carried out using the Gaussian 16 program package [49].

### 2.2. Computational Models

B_27_N_27_H_18_ cluster model was selected to represent the h-BN support; the truncated edges were passivated with hydrogen atoms. The metal atom (M) was introduced at the V_B_ site to form the M@BN (M = Ag, Au) single-atom system. To verify the adequacy of the model size, an extended B_48_N_48_H_24_ cluster was constructed (Figure S1). The metal anchoring configurations at V_B_ sites, as well as the O_2_ adsorption configurations and corresponding energies derived from the large cluster, are nearly identical to those obtained with the original B_27_N_27_H_18_ model (Table S1). These results confirm that the B_27_N_27_H_18_ cluster is sufficiently sized to reliably support the investigation of O_2_ adsorption processes. Carbon was incorporated into the h-BN lattice to replace the lattice B or N atoms, forming C–B and C–N bonds. The metal atom remains anchored at the V_B_ site, forming the M@nC-BN systems. Experiments have confirmed that carbon atoms tend to form atomic domains rather than being atomically dispersed when doped into BN materials [37,39,50,51]. To this end, we selected a series of representative carbon-doped configurations to systematically investigate the effects of doping concentration and structure. The C-doping content was varied between 1 and 8 atoms, corresponding to C contents of 1.5–12.3% for Ag systems and 1.4–11% for Au systems. This range covers the critical region reported in the recent literature, where the bandgap of BCN materials is known to be strongly dependent on C concentration, with an optimal value near 6% leading to the smallest bandgap and highest activity [37]. The possible configurations for 3C and 5C doping are chain-like. As this arrangement type has been thoroughly investigated in even-numbered systems (4C, 6C, 8C), these two cases were therefore not included in the present study.

**Figure 1:**
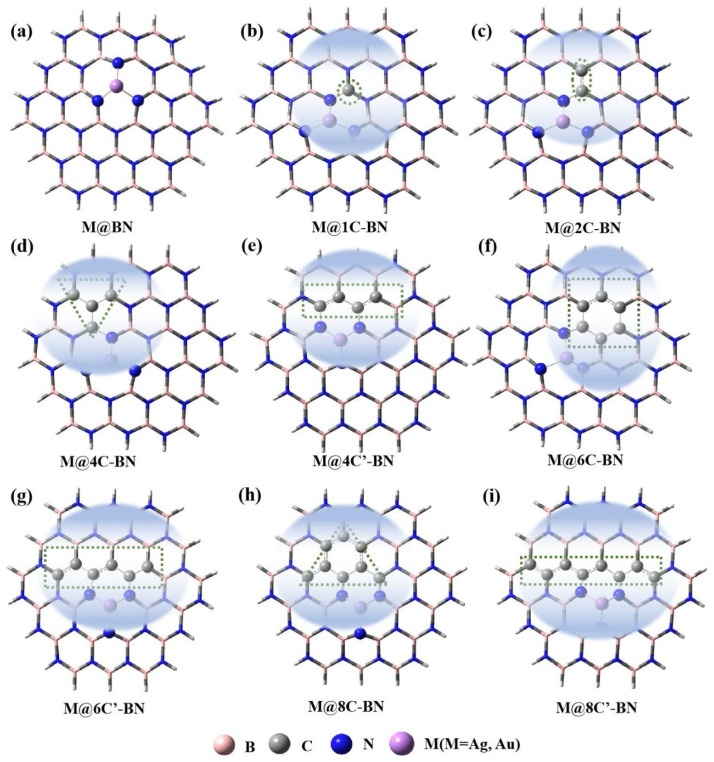
The M@BN (**a**) and M@nC-BN (M = Ag, Au) (**b**–**i**) models.

These configurations, with an increasing number of carbon atoms, are named M@1C-BN, M@2C-BN, M@4C-BN, M@4C′-BN, M@6C-BN, M@6C′-BN, M@8C-BN, and M@8C′-BN, respectively. Here, nC-BN and nC′-BN represent structures with identical carbon atom counts (n) but differing configurations, as illustrated in Figure 1. Specifically, in the C-doped systems, the superscript prime (′) denotes a chain-like configuration of carbon atoms, whereas its absence represents other structures: for n = 4, 4C-BN is triangular and 4C′-BN is chain-like; for n = 6, 6C-BN is ring-like and 6C′-BN is chain-like; for n = 8, 8C-BN is triangular and 8C′-BN is chain-like.

The binding energy (*E*_b_) of M at V_B_ site of BN or BNC support, representing the stability of the metal atom on the surface, is defined by Equation (1)

(1) $$E_{b}=E_{M@\mathrm{support}}-(E_{M}+E_{\mathrm{support}})$$

where $E_{M}$, $E_{\mathrm{support}}$, and $E_{M@\mathrm{support}}$ represent the total energies of the isolated metal atom in the gas phase, the BN or BNC support with a V_B_ site, and the M atom anchored at V_B_ site of BN or BNC systems, respectively. A more negative *E*_b_ value indicates a more stable SAC. Similarly, the adsorption energy (*E*_ads_) of O_2_ on M@support is given by Equation (2):

(2) $$E_{ads}=E_{O_{2}/M@\mathrm{support}}-(E_{O_{2}}+E_{M@\mathrm{support}})$$

where $E_{O_{2}}$, $E_{M@\mathrm{support}}$, and $E_{O_{2}/M@\mathrm{support}}$ denotes the total energies of the ground state O_2_ molecule, the single atom catalyst M@support, and the combined system, respectively. Thus, a negative *E*_ads_ value indicates an exothermic adsorption process. Zero-point energy corrections were applied to all calculated *E*_b_ and *E*_ads_ values.

It has been reported that Au single atoms can exist on the CuO film at 400 K, while Ag single atoms can be anchored on the C_3_N_4_ support surface at 353 K [52,53]. In this study, we also investigated the stability and adsorption properties of Ag and Au single atoms at 400 K by means of frequency calculations. The Gibbs binding energy (*G*_b_) is defined by Equation (3)

(3) $$G_{b}=G_{M@\mathrm{support}}-(G_{M}+G_{\mathrm{support}})$$

with *G*_M_, *G*_support_, and *G*_M@support_ representing their corresponding total Gibbs free energies. Similarly, the Gibbs adsorption energy (*G*_ads_) is given by Equation (4),

(4) $$G_{ads}=G_{O_{2}/M@\mathrm{support}}-(G_{O_{2}}+G_{M@\mathrm{support}})$$

where $G_{O_{2}}$, $G_{M@\mathrm{support}}$, and $G_{O_{2}/M@\mathrm{support}}$ denote their corresponding total Gibbs free energies.

## 3. Results

### 3.1. The Structures and Stability of M@BN and M@nC-BN

**Figure 2:**
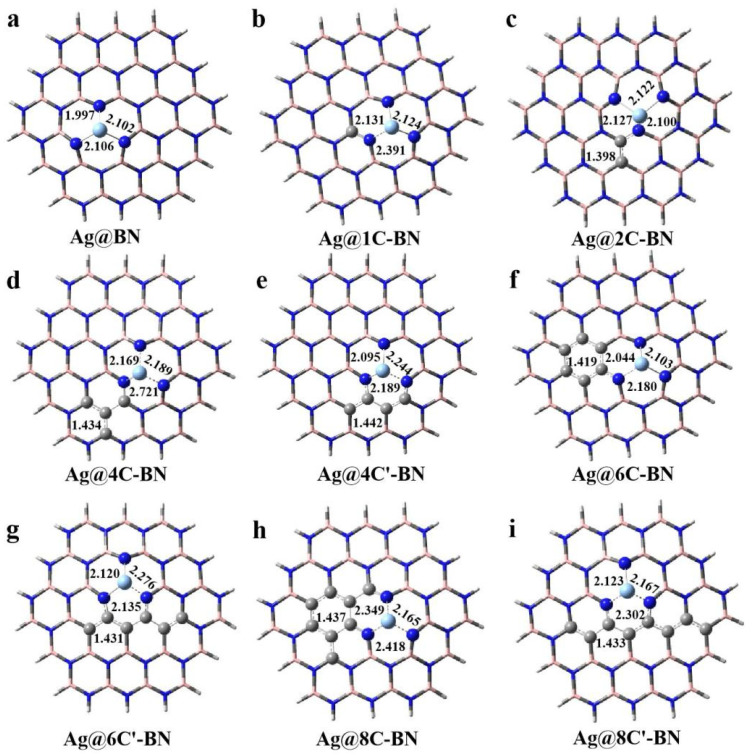
The optimized geometries of Ag anchored at the V_B_ site of h-BN (**a**) or BNC (**b**–**i**) supports.

We first optimized the structures of single Ag and Au anchored on the V_B_ site of h-BN and C-doping BN supports, and the most stable geometries are shown in Figure 2 and Figure S2, respectively. Using the single Ag atom adsorption as an example, in the case of Ag@BN, the Ag atom at the V_B_ site resides out of the BN plane due to its large atomic radius, causing an upward displacement of the adjacent nitrogen atoms. The distances between Ag and the three neighboring N atoms are 1.997, 2.102, and 2.106 Å, respectively, in agreement with values (2.10 Å) reported in the literature [35]. Carbon doping into the h-BN support markedly alters the adsorption configuration of the Ag single atom, primarily marked by the elongation of one of the three initially similar Ag–N bonds. For instance, with one C dopant (n = 1, Figure 2b), one Ag–N bond length increases to 2.391 Å. At n = 8 (in the 8C′-BN structure, Figure 2i), this bond elongates to 2.302 Å. This bond elongation will weaken the binding strength and thus the stability of Ag at the V_B_ site. Similarly, the Au atom at the V_B_ site of the h-BN surface (Figure S2) forms three Au–N bonds (1.970, 2.058, and 2.058 Å), closely matching the literature value of 2.06 Å [54]. Likewise, C doping in the BN support leads to a marked elongation of one Au–N bond. With increasing doping concentration from n = 1 to n = 8, its maximum length rises from 2.080 Å to 2.854 Å, as presented in Figure S2. This substantial elongation weakens the Au-support interaction thereby potentially compromising the stability of the Au single atom at the V_B_ site.

The binding energy (*E*_b_) serves as a direct indicator for evaluating the stability of metal atoms on the support surface, with more negative values corresponding to greater stability of the system. As shown in Table 1, for the M@BN systems, the *E*_b_ of Ag@BN and Au@BN are −61.3 and −62.9 kcal/mol, respectively, which are in good agreement with previous theoretical calculations [55]. The *E*_b_ of Ag is comparable to its cohesive energy (−68.0 kcal/mol). However, the *E*_b_ of Au is less negative than its cohesive energy (−87.8 kcal/mol) [56]. Hence, Ag is more stable than Au when anchored at the V_B_ site of the h-BN surface.

In agreement with the optimized structural results, a slight decrease in the stability of single atoms, is observed upon doping carbon into the BN surface. For instance, when the number of carbon atom equals to 1 (n = 1), the *E*_b_ value of Ag@1C-BN and Au@1C-BN decrease to −52.3 kcal/mol and −47.6 kcal/mol, respectively; when n = 2, the *E*_b_ values of Ag@2C-BN and Au@2C-BN decrease to −41.6 kcal/mol and −44.0 kcal/mol, respectively. With an increasing number of doped carbon atoms, the configuration of carbon doping in the BN surface significantly influences the stability of single atoms. When the carbon dopant count reaches 4, 6, and 8, the two configurations exhibit distinct stability: the triangular configuration consistently demonstrates higher stability than the corresponding chain-like structure, and Ag@nC-BN systems show greater stability than their Au@nC-BN counterparts. Moreover, in the Ag single-atom system, Ag@4C-BN exhibits the highest stability with a binding energy of −52.6 kcal/mol, thus representing the most stable C-doped Ag single-atom configuration. For the C-doped Au single-atom system, Au@6C-BN shows the best stability with a binding energy of −49.8 kcal/mol. The electronic structure, which originates from orbital hybridization between the C 2p and metal d orbitals near the HOMO level, is the primary driving force for stabilizing the M@BN-6C single-atom systems, as evidenced by the density of states (DOS) plots (Figure S3). For Au, relativistic effects cause contraction of the s orbital and expansion of the d/f orbitals, which further enhances the orbital overlap with the C 2p orbital, as shown in the HOMO diagram in Figure S4. This intensified d-p orbital interaction directly contributes to the greater stability exhibited by the Au@6C-BN system.

At 400 K, the stability of Ag and Au single atoms decreased, as evidenced by the *G*_b_ values in Table 1, while the stability order remained unchanged. Metal atoms anchored on the undoped BN support exhibited the highest stability, outperforming all C-doped counterparts. Among the C-doped systems, Ag and Au single atoms displayed optimal stability on the 4C-BN and 6C-BN supports, respectively.

In summary, Ag forms more stable SACs than Au at the V_B_ site of BN support. Carbon doping elongates the M–N bonds, thereby reducing metal stability. Both the amount and arrangement of doped C atoms affect stability. Chain-like configurations show a strong influence with more C atoms, while triangular (4C-BN) and ring (6C-BN) ones have little effect. Ag@4C-BN and Au@6C-BN are the most stable among all configurations.

### 3.2. Adsorption of Triplet O_2_ on M@BN and M@nC-BN

**Figure 3:**
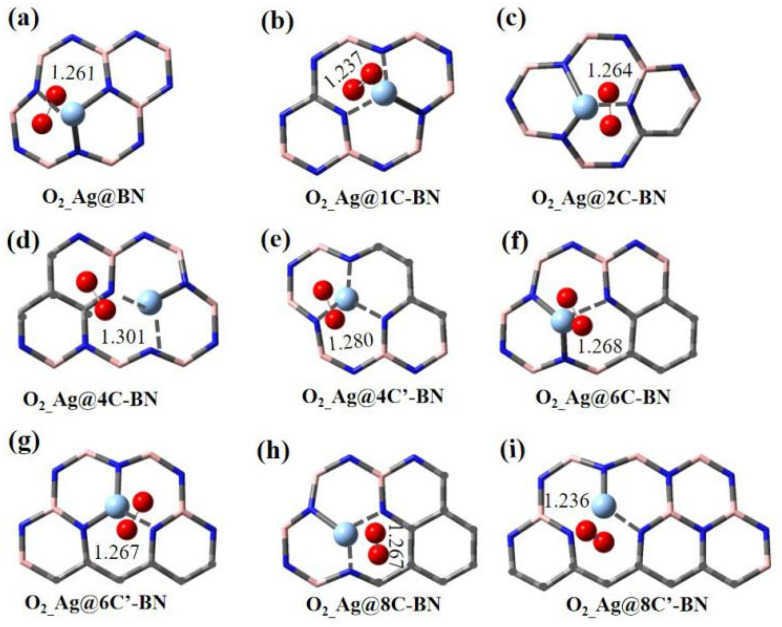
Optimized adsorption configurations of triplet O_2_ on Ag@BN (**a**) and Ag@nC-BN (**b**–**i**), only section of h-BN is displayed.

Given the triplet ground state of O_2_, the adsorption behavior of triplet O_2_ on the surfaces of Ag and Au single atoms was first investigated, with the corresponding configurations shown in Figure 3 and Figure S5, respectively. For O_2_ adsorbed on Ag SACs, the O–O bond length ranges from 1.236 to 1.280 Å across most systems, except for 4C-BN (1.301 Å). On Au SACs, the longest O–O bond lengths occur on Au@4C′-BN and Au@8C′-BN. However, due to the poor stability of Au single atoms in these systems, they are not considered ideal for catalysis when stability is taken into account.

The *E*_ads_ of triplet O_2_ are listed in Table S2. All adsorption energies are negative values, indicating that the adsorption process is spontaneous. Among these systems, M@2C-BN system exhibits the strongest adsorption toward O_2_, with *E*_ads_ of −27.2 kcal/mol and −31.3 kcal/mol for Ag@2C-BN and Au@2C-BN, respectively. Even at 400 K, when *G*_ads_ decreases on all M@nC-BN systems, M@2C-BN still maintains the strongest adsorption capacity.

The activation of O_2_ typically requires a spin-state transition from triplet to singlet, a process often governed by non-adiabatic charge transfer and surface electronic coupling on metal-based catalysts [57,58]. This transition overcomes the spin-forbidden constraint for O_2_ activation. For instance, Behler et al. observed a triplet-to-singlet transition during O_2_ adsorption on Al(001) via charge transfer-induced spin population reduction [59]; Kurokawa et al. reported a triplet-quintet-singlet transition in hemoglobin-O_2_ binding that accelerates reactions [57]; Belanzoni et al. confirmed that O–O bond cleavage requires a low-spin state transition of Fe(II)/EDTA-O_2_ complexes [58]. Given this, we further investigated the adsorption behavior of singlet O_2_.

### 3.3. Adsorption of Singlet O_2_ on M@BN and M@nC-BN

#### 3.3.1. Singlet O_2_ Adsorption on Ag@BN and Ag@nC-BN

**Figure 4:**
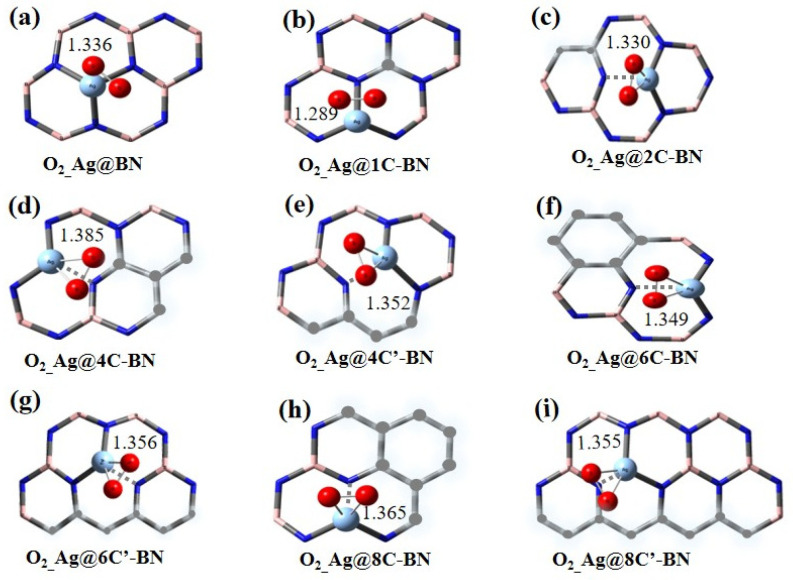
Optimized adsorption configurations of singlet O_2_ on Ag@BN (**a**) and Ag@nC-BN (**b**–**i**), only section of h-BN is displayed.

Figure 4 shows the optimized adsorption configurations of singlet O_2_ on Ag single-atom catalysts. For O_2_ adsorption on Ag@BN, the O–O bond length elongates from 1.19 Å in the gas phase to 1.336 Å. Upon adsorption on Ag@nC-BN surfaces, the O–O bond lengths show significant increases compared to the free molecule. Notably, the O–O bond elongates more significantly in the singlet than in the triplet adsorption configuration, indicating that the adsorbed singlet oxygen is more readily activated. Moreover, the elongation is most pronounced after O_2_ adsorption on Ag@4C-BN (Figure 4d), where the bond length reaches 1.385 Å, indicating the strongest interaction between O_2_ and Ag@4C-BN.

**Figure 5:**
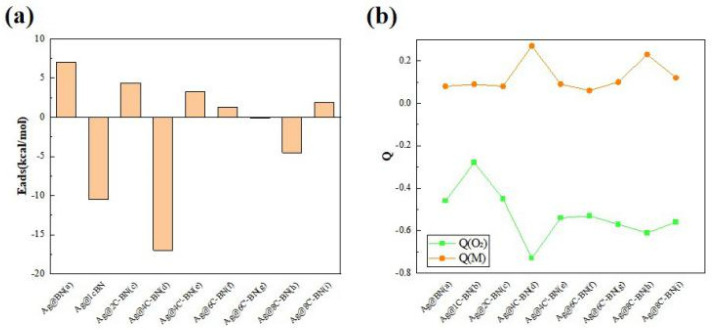
(**a**) Adsorption energies (*E*_ads_, kcal/mol) and (**b**) charges for singlet O_2_ adsorption on Ag single-atom catalysts.

The adsorption energy (*E*_ads_) directly reflects the strength of the interaction between O_2_ and the catalysts. Specifically, a more negative *E*_ads_ value signifies a stronger adsorption interaction. The *E*_ads_ and charge analysis for O_2_ on Ag SACs are presented in Figure 5 and Table S3. The positive *E*_ads_ of O_2_ on the Ag@BN surface is 7.0 kcal/mol, indicating a weak interaction. When carbon is doped into the BN support, the adsorption strength of singlet O_2_ on the catalyst surfaces increases. For instance, on the Ag@1C-BN surface, the adsorption energy becomes negative (−10.5 kcal/mol). The strongest adsorption occurs on the Ag@4C-BN surface, with an *E*_ads_ of −17.0 kcal/mol. Therefore, doping the support with carbon atoms effectively modulates the catalyst’s activity. Notably, At 400 K, *G*_ads_ of O_2_ becomes more positive across all systems, while the strongest O_2_ adsorption is still maintained on the Ag@4C-BN surface.

Furthermore, the geometric arrangement of the C-doping is also a crucial factor affecting O_2_ adsorption performance, especially for systems with four and eight carbon atoms. For n = 4, the O_2_ adsorption energy differs significantly between the Ag@4C-BN (−17.0 kcal/mol) and Ag@4C′-BN (3.3 kcal/mol) configurations. This trend persists with 8C-doped systems, where the energies are −4.5 kcal/mol for Ag@8C-BN and 1.9 kcal/mol for Ag@8C′-BN, respectively.

Natural population analysis further corroborates the interaction strength between oxygen and the catalysts. After O_2_ adsorption, the molecule acquires a negative charge and the Ag atom becomes more positive, indicating electron transfer from the metal to the adsorbate. This transfer populates the O_2_ antibonding 2π* orbital, which consequently weakens the O–O bond, as evidenced by a significant increase in bond length and the resultant activation of O_2_. Taking O_2_ adsorption on Ag@4C-BN as an example, the charge on O_2_ is −0.73 |e|, indicating substantial activation of O_2_.

A comparison between the Ag@BN and Ag@4C-BN systems reveals a clear trade-off between the stability and catalytic activity. On the Ag@BN surface, where the Ag atom is most stable (*E*_b_ = −61.3 kcal/mol), the O_2_ adsorption is weakest (*E*_ads_ = 7.0 kcal/mol). Conversely, when the metal-support interaction is slightly weakened, as on the Ag@4C-BN surface (*E*_b_ = −52.6 kcal/mol), O_2_ adsorption becomes significantly stronger (*E*_ads_= −17.0 kcal/mol), indicating strong chemisorption. In the following section, we will elaborate on the activity difference between M@BN and M@4C-BN by analyzing their electronic structures.

#### 3.3.2. Singlet O_2_ Adsorption on Au@BN and Au@nC-BN

**Figure 6:**
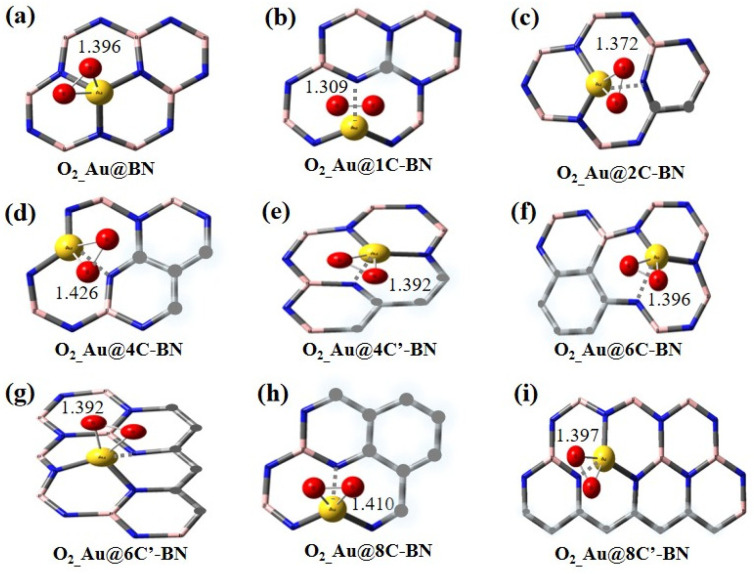
Optimized adsorption configurations of singlet O_2_ on Au single-atom catalysts, only section of h-BN is displayed Au@BN (**a**) and Au@nC-BN (**b**–**i**).

Figure 6 Shows the optimized adsorption configurations of singlet O_2_ on Au single-atom catalysts. After O_2_ adsorption, the O–O bond length elongates from 1.19 Å to 1.396 Å on Au@BN surface. Adsorption on Au@nC-BN surfaces also leads to significant O–O bond elongation compared to the free molecule. Notably, the elongation is most pronounced after O_2_ adsorption on Au@4C-BN (Figure 6d), where the bond length reaches 1.426 Å, approaching the O_2_^2−^ bond length (1.49 Å). In line with the Ag-based systems, the O–O bond of adsorbed singlet O_2_ on the most of Au-based systems elongates more than that of triplet adsorbed O_2_, indicating stronger activation of singlet adsorbed O_2_.

**Figure 7:**
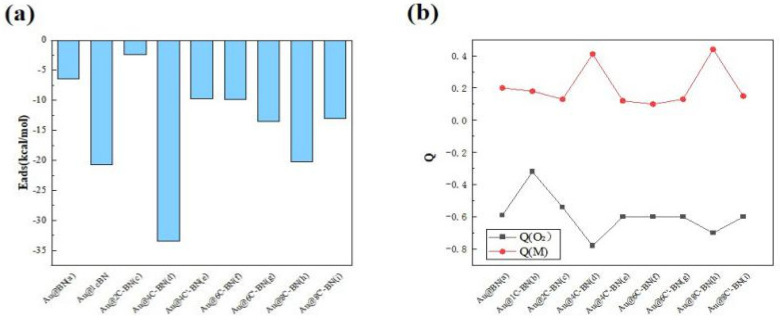
(**a**) Adsorption energies (*E*_ads_, kcal/mol) and (**b**) charges analysis for singlet O_2_ adsorption on Au single-atom catalysts.

As presented in Figure 7 and Table S4, the O_2_ adsorption energy on the Au@BN surface is only −6.4 kcal/mol, characteristic of weak physisorption. Apart from Au@2C-BN, O_2_ adsorption energies become more negative on all C-doped support surfaces. Notably, the strongest O_2_ adsorption is observed on Au@4C-BN, with an *E*_ads_ of −33.4 kcal/mol. Charge analysis indicates that the process is activated by electron transfer to the O_2_ molecule, consistent with the behavior seen on Ag single atoms. The transferred electrons occupy the antibonding orbital of O_2_, leading to its activation. As a case in point, the O_2_ molecule on Au@4C-BN carries the highest charge of −0.78|e|.

The carbon doping geometry also influences O_2_ adsorption, with the triangular configuration (Au@4C-BN) demonstrating significantly stronger adsorption (−33.4 kcal/mol) than the chain-like form (Au@4C′-BN, −9.8 kcal/mol) for n = 4. A trend that holds for n = 8, −20.3 vs. −13.0 kcal/mol), confirming the superior promotion of O_2_ activation by the triangular structure.

In summary, carbon doping universally enhance singlet O_2_ adsorption on Ag and Au catalysts, with triangular configurations being most effective. Within the M@4C-BN systems (M = Ag, Au), O_2_ demonstrates greater O–O bond elongation and a higher degree of activation than the triplet state, highlighting the importance of spin state in tuning catalytic performance. These findings confirm M@4C-BN as a potent platform for facilitating O_2_ activation.

#### 3.3.3. Electron Density Difference (EDD) Analysis

**Figure 8:**
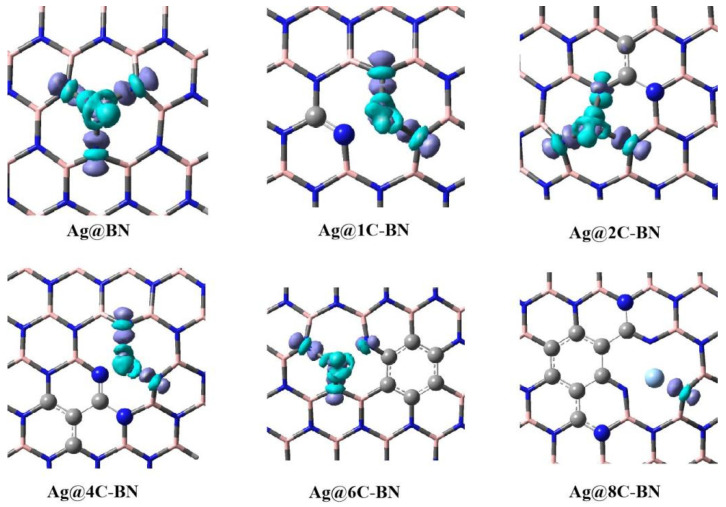
Electron density difference (EDD) plots for Ag@BN and Ag@nC-BN systems (Isosurface = 0.02 a.u.).

The concentration and configuration of carbon doping are closely related to the stability and activity of SACs. Introducing carbon atoms into the support slightly reduces the stability of the single atoms but enhances the catalyst’s reactivity towards O_2_ activation. This is likely intimately connected to the changes in the electronic structure of the support induced by carbon doping. Figure 8 and Figure S6 presents the electron density difference (EDD) plots for the Ag and Au single-atom systems, respectively. Regions in purple indicate an increase in electron density, while cyan/green regions indicate a decrease. In the Ag@BN system, electron density depletion occurs primarily around the metal atom, and density accumulation is observed between Ag and the three surrounding N atoms, indicating the formation of three stable Ag–N covalent bonds, which explains the good stability of the Ag@BN system. As carbon atoms are doped into the BN surface, the regions of electron density accumulation between Ag and N decrease significantly, leading to reduced stability of the corresponding catalysts. When the coordination ability between the metal atom and N weakens, the metal atom possesses more free electrons available for coordination with adsorbates, which may account for the observed increase in reactivity [33].

**Figure 9:**
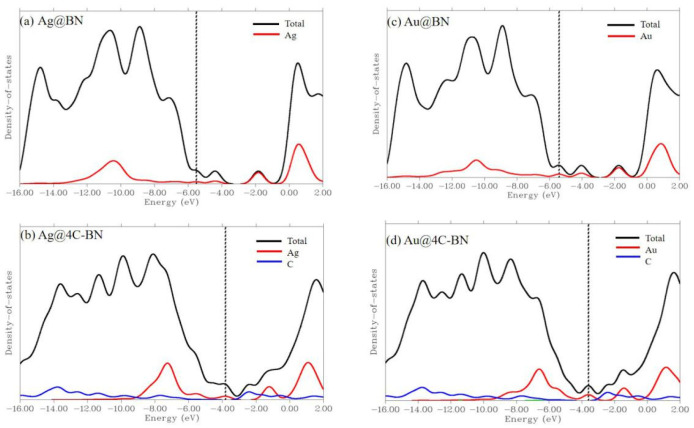
Total density of states (TDOS) and partial density of states (PDOS).

To further clarify the enhanced O_2_ reactivity of M@4C-BN, we plotted the density of states (DOS) and frontier orbital diagrams of M@BN and M@4C-BN (Figure 9 and Figure S7, respectively). DOS analysis reveals that carbon doping rearranges the electron distribution of M@BN. Specifically, the C 2p orbitals hybridization effectively elevate the HOMO energy level of M@4C-BN to −3.8 eV (Ag) and −3.6 eV (Au), substantially higher than that of M@BN (−5.5 eV). A higher HOMO level is generally associated with a stronger electron-donating tendency [60,61], facilitating electron transfer to the antibonding orbital of adsorbed O_2_ and thus enhancing reactivity.

Therefore, the concentration and geometric configuration of carbon doping are central factors influencing the stability and activity of metal single-atom catalysts. This conclusion provides a theoretical basis for optimizing h-BN-based single-atom catalysts in catalytic oxidation applications.

### 3.4. The Dissociation of Singlet O_2_ on M@BN and M@nC-BN

In view of the fact that adsorbed singlet O_2_ on the M@4C-BN surface shows the most significant O–O bond activation with a bond length comparable to that of O_2_^2−^, we further investigated the dissociation process of O_2_ on this surface, the corresponding configurations and energies during the dissociation process are presented in Figure S8. The adsorbed singlet O_2_ as the reaction entrance, in which the O–O bond lengths (1.385 Å on Ag SAC, 1.426 Å on Au SAC). Through a transition state, the O–O bond elongates to 2.769 Å (Ag) and 2.547 Å (Au) before fully dissociating, reaching final lengths of 3.160 Å (Ag) and 3.057 Å (Au). Energetically, complete dissociation faces high barriers, 86.8 kcal/mol on Ag SAC and 66.6 kcal/mol on Au SAC, relative to the adsorbed O_2_ state. The overall process is also endothermic by 79.7 kcal/mol (Ag) and 55.5 kcal/mol (Au), confirming its thermodynamic and kinetic difficulty. Notably, our previous work has verified that the adsorbed singlet O_2_ itself can act as reactive oxygen species to participate in dibenzothiophene oxidation reactions [34,48]. Moreover, for CO catalytic oxidation, the direct oxidation pathway involving activated molecular oxygen appears to be the predominant mechanism [44]. Therefore, the reactive oxygen species formed via adsorption probably play a crucial role in facilitating efficient oxidation reactions.

## 4. Conclusions

Using density functional theory (DFT), this work investigates the adsorption and activation of O_2_ on single-atom catalysts (M@BN, M@BNC, M=Ag, Au), to elucidate the critical influence of carbon doping in the support on the catalytic stability and oxidative activity. The main conclusions are as follows:

(1) Carbon doping reduces Ag and Au single-atom catalysts stability by weakening the metal-substrate bond. This destabilization is severe for chain-like dopant arrangements but minimal for triangular (4C-BN) and ring (6C-BN) configurations, leading to the Ag@4C-BN and Au@6C-BN as the most stable systems.
(2) From the perspective of geometric configurations, adsorbed singlet O_2_ achieves more efficient O–O bond activation than its triplet counterpart. Notabaly, the geometry of carbon dopants critically governs singlet O_2_ adsorption on M@BNC systems. A triangular doping arrangement, dramatically enhances adsorption and O–O bond activation compared to undoped M@BN. This is most evident for Ag@4C-BN and Au@4C-BN, which exhibit maximal adsorption energies of −17.0 and −33.4 kcal/mol, respectively.
(3) The density of states (DOS) and frontier orbital analysis reveal that when carbon is doped in a triangular configuration, its 2p orbitals significantly contribute to the HOMO energy level of M@4C-BN, causing a notable upward shift. This shift facilitates the transfer of electrons to the antibonding orbitals of the adsorbed O_2_, thereby effectively promoting O_2_ activation.

## Figures and Tables

**Table 1:** Binding energies (*E*_b_, kcal/mol) and Gibbs binding energies (*G*_b_, kcal/mol) at 400 K for M@BN and M@nC-BN (M = Ag, Au).

Model	M@BN (a)	M@1C-BN (b)	M@2C-BN (c)	M@4C-BN (d)	M@4C′-BN (e)	M@6C-BN (f)	M@6C′-BN (g)	M@8C-BN (h)	M@8C′-BN (i)
*E*_b,Ag_	−61.3	−52.3	−41.6	−52.6	−40.1	−50.0	−40.3	−50.0	−39.0
*E*_b,Au_	−62.9	−47.6	−44.0	−45.3	−36.5	−49.8	−34.3	−40.9	−32.3
*G*_b,Ag_	−46.5	−39.5	−26.3	−40.1	−28.3	−34.8	−24.7	−36.2	−26.7
*G*_b,Au_	−48.1	−31.7	−28.8	−32.5	−23.7	−34.0	−18.6	−27.1	−19.4

## Data Availability

The original contributions presented in this study are included in the article/Supplementary Material. Further inquiries can be directed to the corresponding author.

## Supplementary Materials

The following supporting information can be downloaded at: https://www.mdpi.com/article/10.3390/nano16010061/s1, Figure S1. The larger models for (a) Ag@BN, (b) Au@BN; O_2_ adsorption geometries on these SACs: (c) O_2__Ag@BN, (d) O_2__Au@BN. Figure S2. The optimized geometries of Au anchored at the VB site of BN (a) or BNC (b–i) supports. Figure S3. The total density of states (DOS) and partial density of states (PDOS) of (a) Ag@4C-BN, (b) Ag@6C-BN, (c) Au@4C-BN, and (d) Au@6C-BN. Figure S4. The HOMO diagrams of (a) Ag@6C-BN and (b) Au@6C-BN (isosurface value = 0.02 a.u.). Figure S5. Optimized adsorption configurations of triplet O_2_ on Au@BN and Au@nC-BN, only section of h-BN is displayed. Figure S6. Electron density difference (EDD) plots for Au@BN and Au@nC-BN systems (Isosurface value= 0.02 a.u.). Figure S7. HOMO and LUMO energy level diagrams for M@BN (a) and M@4C-BN (b) systems. Figure S8. Optimized geometries of O_2_ dissociation on (a) Ag@4C-BN, (b) Au@4C-BN, and (c) the energy profile along O_2_ dissociation process. Table S1. Binding Energy (*E*_b_) of M@BN (M= Ag, Au) and adsorption energy (*E*_ads_) of O_2_ on original and larger Models (unit: kcal/mol). Table S2. Adsorption energies (*E*_ads_, kcal/mol), adsorption Gibbs free energies (*G*_ads_, kcal/mol) for triplet O2 on Ag and Au single-atom catalysts. Table S3. Adsorption energies (*E*_ads_, kcal/mol), adsorption Gibbs free energies (*G*_ads_, kcal/mol) and charge analysis for singlet O_2_ adsorption on Ag single-atom catalysts. Table S4. Adsorption energies (*E*_ads_, kcal/mol), adsorption Gibbs free energies (*G*_ads_, kcal/mol) and charge analysis for singlet O_2_ adsorption on Au single-atom catalysts.

## References

[1] Kaiser S.K., Chen Z., Akl D.F., Mitchell S., Pérez-Ramírez J. (2020). Single-atom catalysts across the periodic table. Chem. Rev..

[2] Li Z., Wei W., Li H., Li S., Leng L., Zhang M., Horton J.H., Wang D., Sun W., Guo C. (2021). Low-temperature synthesis of single palladium atoms supported on defective hexagonal boron nitride nanosheet for chemoselective hydrogenation of cinnamaldehyde. ACS Nano.

[3] Li Z., Wang D., Wu Y., Li Y. (2018). Recent Advances in the Precise Control of Isolated Single-Site Catalysts by Chemical Methods. Natl. Sci. Rev..

[4] Hu Y., Dou Y., Sun Z., Li B., Zhou H., Wang S., Hu X., He K., Qu M., Chen W. (2022). Rational Design, Application and Dynamic Evolution of Cu-N-C Single Atom Catalyst. J. Mater. Chem. A.

[5] Zhang H., Liu G., Shi L., Ye J. (2017). Single-Atom Catalysts: Emerging Multifunctional Materials in Heterogeneous Catalysis. Adv. Energy Mater..

[6] Cheng Q., Yang L., Zou L., Zou Z., Chen C., Hu Z., Yang H. (2017). Single cobalt atom and N codoped carbon nanofibers as highly durable electrocatalyst for oxygen reduction reaction. ACS Catal..

[7] Li X., Bi W., Zhang L., Tao S., Chu W., Zhang Q., Luo Y., Wu C., Xie Y. (2016). Single-atom Pt as Co-Catalyst for enhanced photocatalytic H_2_ evolution. Adv. Mater..

[8] Liu P., Zhao Y., Qin R., Mo S., Chen G., Gu L., Chevrier D.M., Zhang P., Guo Q., Zang D. (2016). Photochemical route for synthesizing atomically dispersed palladium catalysts. Science.

[9] Qiao B., Lin J., Wang A., Chen Y., Zhang T., Liu J. (2015). Highly active Au_1_/Co_3_O_4_ single-atom catalyst for CO oxidation at room temperature. Chin. J. Catal..

[10] Jiang R., Li L., Sheng T., Hu G., Chen Y., Wang L. (2018). Edge-Site Engineering of Atomically Dispersed Fe–N4 by Selective C–N Bond Cleavage for Enhanced Oxygen Reduction Reaction Activities. J. Am. Chem. Soc..

[11] Poh C.K., Lim S.H., Lin J., Feng Y.P. (2014). Tungsten carbide supports for single-atom platinum-based fuel-cell catalysts: First-principles study on the metal–support interactions and O_2_ dissociation on W_x_C low-index surfaces. J. Phys. Chem. C.

[12] Zhou B., Liu K., Yu K., Zhou Q., Gao Y., Gao X., Chen Z., Chen W., Chen P. (2025). Ultrafast synthesis of single-atom catalysts for electrocatalytic applications. Small.

[13] Lamichhane B., Lee D., Gautam B., Ponce A., Xu F., Kattel S. (2025). The selection of stable Pt single-atom catalysts supported on 3d–5d transition metal carbides. Chem. Mater..

[14] Loy A.C.M., Teng S.Y., How B.S., Zhang X., Cheah K.W., Butera V., Leong W.D., Chin B.L.F., Yiin C.L., Taylor M.J. (2023). Elucidation of single atom catalysts for energy and sustainable chemical production: Synthesis, characterization and frontier science. Prog. Energy Combust. Sci..

[15] Lu Y., Zhang Z., Wang H., Wang Y. (2021). Toward efficient single-atom catalysts for renewable fuels and chemicals production from biomass and CO_2_. Appl. Catal. B Environ..

[16] Mahak D., Vivek P. (2018). Supported single atom and pseudo-single atom of metals as sustainable heterogeneous nanocatalyst. ChemCatChem.

[17] Zeng L., Cheng K., Sun F., Fan Q., Li L., Zhang Q., Wei Y., Zhou W., Kang J., Zhang Q. (2024). Stable anchoring of single rhodium atoms by indium in zeolite alkane dehydrogenation catalysts. Science.

[18] Gul S., Nasim F., Nadeem M.A. (2025). Single-atom metal-based electrocatalysts (Fe, Ni, and Co) for CO_2_-to-CO Conversion: A Comprehensive Review. ACS Appl. Energy Mater..

[19] Yan H., Cheng H., Yi H., Lin Y., Yao T., Wang C., Li J., Wei S., Lu J. (2015). Single-atom Pd_1_/Graphene catalyst achieved by atomic layer deposition: Remarkable performance in selective hydrogenation of 1,3-butadiene. J. Am. Chem. Soc..

[20] Guo Y., Liang J., Huang Y., Yang J., Zhang Q., Wang A., Qiao B., Li J., Zhang T. (2025). Covalent and strong metal–support interactions for robust single-atom catalysts. Acc. Chem. Res..

[21] Weng Q., Wang X., Wang X., Bandoa Y., Golberg D. (2016). Functionalized hexagonal boron nitride nanomaterials: Emerging properties and applications. Chem. Soc. Rev..

[22] Zhou C., Lai C., Zhang C., Zeng G., Huang D., Cheng M., Hu L., Xiong W., Chen M., Wang J. (2018). Semiconductor/boron nitride composites: Synthesis, properties, and photocatalysis applications. Appl. Catal. B Environ..

[23] Roy S., Zhang X., Puthirath A.B., Meiyazhagan A., Bhattacharyya S., Rahman M.M., Babu G., Susarla S., Saju S.K., Tran M.K. (2021). Structure, properties and applications of two-dimensional hexagonal boron nitride. Adv. Mater..

[24] Rajendran A., Fan H.-X., Feng J., Li W.-Y. (2020). Desulfurization on boron nitride and boron nitride-based materials. Chem.-Asian J..

[25] Wang C., Chen Z., Yao X., Chao Y., Xun S., Xiong J., Fan L., Zhu W., Li H. (2018). Decavanadates anchored into micropores of graphene-like boron nitride: Efficient heterogeneous catalysts for aerobic oxidative desulfurization. Fuel.

[26] Wu P., Ma S., Zhou S., Sun Y., Chen L., Liu J., Zhu W., Xu C. (2025). Fe_3_N nanoparticles confined in h-BN for Ultra-Deep oxidative desulfurization using molecular oxygen. Chem. Eng. Sci..

[27] Zhang Y., Du H., Ma Y., Ji L., Guo H., Tian Z., Chen H., Huang H., Cui G., Asiri A.M. (2019). Hexagonal boron nitride nanosheet for effective ambient N_2_ fixation to NH_3_. Nano Res..

[28] Wong D., Velasco J., Ju L.L.J., Kahn S., Tsai H.-Z., Germany C., Taniguchi T., Watanabe K., Zettl A., Wang F. (2015). Characterization and manipulation of individual defects in insulating hexagonal boron nitride using scanning tunnelling microscopy. Nat. Nanotechnol..

[29] Meyer J.C., Chuvilin A., Algara-Siller G., Biskupek J., Kaiser U. (2009). Selective sputtering and atomic resolution imaging of atomically thin boron nitride membranes. Nano Lett..

[30] Du A., Chen Y., Zhu Z., Amal R., Lu G.Q., Smith S. (2009). Dots versus antidots: Computational exploration of structure, magnetism, and half-metallicity in boron-nitride nanostructures. J. Am. Chem. Soc..

[31] Jin C., Lin F., Suenaga K., Iijima S. (2009). Fabrication of a freestanding boron nitride single layer and its defect assignments. Phys. Rev. Lett..

[32] Li D., Li W., Zhang J. (2020). Fe doped BN monolayer: A promising low-cost single atom catalyst for promoted CO oxidation activity. Appl. Surf. Sci..

[33] Datta J., Majumder C. (2021). Stabilizing Co, Ni and Cu on the h-BN surface: Using O–O bond activation to probe their performance as single atom catalyst. Catal. Today.

[34] Lv N., Ran H., Zhang J., Yin J., Zhang Y., Li H., Zhu L. (2024). The single metal atom (Ni, Pd, Pt) anchored on defective hexagonal boron nitride for oxidative desulfurization. Phys. Chem. Chem. Phys..

[35] Lu Z., Lv P., Yang Z., Li S., Ma D., Wu R. (2017). A promising single atom catalyst for CO oxidation: Ag on boron vacancies of h-BN sheets. Phys. Chem. Chem. Phys..

[36] Sun P.-F., Wang W.-L., Zhao X., Dang J.-S. (2020). Defective h-BN sheet embedded atomic metals as highly active and selective electrocatalysts for NH_3_ fabrication via NO reduction. Phys. Chem. Chem. Phys..

[37] Fan M., Wu J., Yuan J., Deng L., Zhong N., He L., Cui J., Wang Z., Behera S.K., Zhang C. (2019). Doping nanoscale graphene domains improves magnetism in hexagonal boron nitride. Adv. Mater..

[38] Huang C., Chen C., Zhang M., Lin L., Ye X., Lin S., Antonietti M., Wang X. (2015). Carbon-doped BN nanosheets for metal-free photoredox catalysis. Nat. Commun..

[39] Ci L., Song L., Jin C., Jariwala D., Wu D., Li Y., Srivastava A., Wang Z.F., Storr K., Balicas L. (2010). Atomic layers of hybridized boron nitride and graphene domains. Nat. Mater..

[40] Zhang Y., Ran H., Liu X., Zhang X., Yin J., Zhang J., He J., Li H., Li H. (2023). Cu-doped BCN nanofibers for highly selective adsorption desulfurization through S-Cu coordination and π-π interaction. Sep. Purif. Technol..

[41] Wang J., Luo X. (2024). Theoretical investigation of the BCN monolayer and their derivatives for metal-free CO_2_ photocatalysis, capture, and utilization. ACS Omega.

[42] Ma C., Zhang Y., Yan S., Liu B. (2022). Carbon-doped boron nitride nanosheets: A high-efficient electrocatalyst for ambient nitrogen reduction. Appl. Catal. B Environ..

[43] Haruta M. (2005). Catalysis—Gold rush. Nature.

[44] Haruta M., Daté M. (2001). Advances in the catalysis of Au nanoparticles. Appl. Catal. A.

[45] Lou Y., Cai Y., Hu W., Wang L., Dai Q., Zhan W., Guo Y., Hu P., Cao X.-M., Liu J. (2020). Identification of active area as active center for CO oxidation over single Au atom catalyst. ACS Catal..

[46] Lyalin A., Nakayama A., Uosaki K., Taketsugu T. (2014). Adsorption and catalytic activation of the molecular oxygen on the metal supported h-BN. Top. Catal..

[47] Andrae D., HauBermann U., Dolg M., Stoll H., PreuB H. (1990). Energy-adjusted ab initio pseudopotentials for the second and third row transition elements. Theor. Chem. Acc..

[48] Lv N., Zhang J., Yin J., Ran H., Zhang Y., Zhu T., Li H. (2022). Atomic Cu and Ni anchoring on h-BN for O_2_ activation and subsequent oxidative desulfurization. Catal. Commun..

[49] Frisch M.J., Trucks G.W., Schlegel H.B., Scuseria G.E., Robb M.A., Cheeseman J.R., Scalmani G., Barone V., Petersson G.A., Nakatsuji H. (2016). Gaussian 16.

[50] Krivanek O.L., Chisholm M.F., Nicolosi V., Pennycook T.J., Corbin G.J., Dellby N., Murfitt M.F., Own C.S., Szilagyi Z.S., Oxley M.P. (2010). Atom-by-atom structural and chemical analysis by annular dark-field electron microscopy. Nature.

[51] Wang Y., Meng J., Tian Y., Chen Y., Wang G., Yin Z., Jin P., You J., Wu J., Zhang X. (2020). Deep ultraviolet photodetectors based on carbon-doped two dimensional hexagonal boron nitride. ACS Appl. Mater. Interfaces.

[52] Zhou X., Shen Q., Yuan K., Yang W., Chen Q., Geng Z., Zhang J., Shao X., Chen W., Xu G. (2018). Unraveling charge state of supported Au single-atoms during CO oxidation. J. Am. Chem. Soc..

[53] Yu H., Xiao H., Yu Z., Chen F., Li W., Qi L., Zhu F., Zhu B., Zhang S., Zhao Z. (2024). Optimization of Ag single atom dispersed graphitic carbon nitride forenhanced catalytic performance in the degradation of rhodamine B and tetracycline. Mater. Res. Bull..

[54] Gao M., Lyalin A., Taketsugu T. (2012). Catalytic activity of Au and Au_2_ on the h-BN surface: Adsorption and activation of O_2_. J. Phys. Chem. C.

[55] Lin S., Ye X., Johnson R., Guo H. (2013). First-principles investigations of metal (Cu, Ag, Au, Pt, Rh, Pd, Fe, Co and Ir) doped hexagonal boron nitride nanosheets: Stability and catalysis of CO oxidation. J. Phys. Chem. C.

[56] Yang X., Song W., Liao K., Wang X., Wang X., Zhang J., Wang H., Chen Y., Yan N., Han X. (2024). Cohesive energy discrepancy drives the fabrication of multimetallic atomically dispersed materials for hydrogen evolution reaction. Nat. Commun..

[57] Kurokawa D., Gueriba J.S., Dino W.A. (2018). Spin-dependent O_2_ binding to hemoglobin. ACS Omega.

[58] Belanzoni P., Bernasconi L., Baerends E.J. (2009). O_2_ activation in a dinuclear Fe(II)/EDTA complex: Spin surface crossing as a route to highly reactive Fe(IV)oxo species. J. Phys. Chem. A.

[59] Behler J., Reuter K., Scheffler M. (2008). Nonadiabatic effects in the dissociation of oxygen molecules at the Al (111) surface. Phys. Rev. B.

[60] Kärkäs M.D., Verho O., Johnston E.V., Åkermark B. (2014). Artificial photosynthesis: Molecular systems for catalytic water oxidation. Chem. Rev..

[61] Jiang R., Wu X., Liu H., Guo J., Zou D., Zhao Z., Tang B.Z. (2021). High-performance orange–red organic light-emitting diodes with external quantum efficiencies reaching 33.5% based on carbonyl-containing delayed fluorescence molecules. Adv. Sci..

