# Supplementary material for: Tuning the Oxidative Activity of Single Atom Catalysts by Carbon Doping in Hexagonal Boron Nitride Supports
Source: Nanomaterials (Basel). 2025 Dec 31;16(1):61. doi: 10.3390/nano16010061 (PMC12787476; doi:10.3390/nano16010061)
Supplement: Supplementary file 1 [file nanomaterials-16-00061-s001.zip › nanomaterials-4031630-supplementary.pdf]

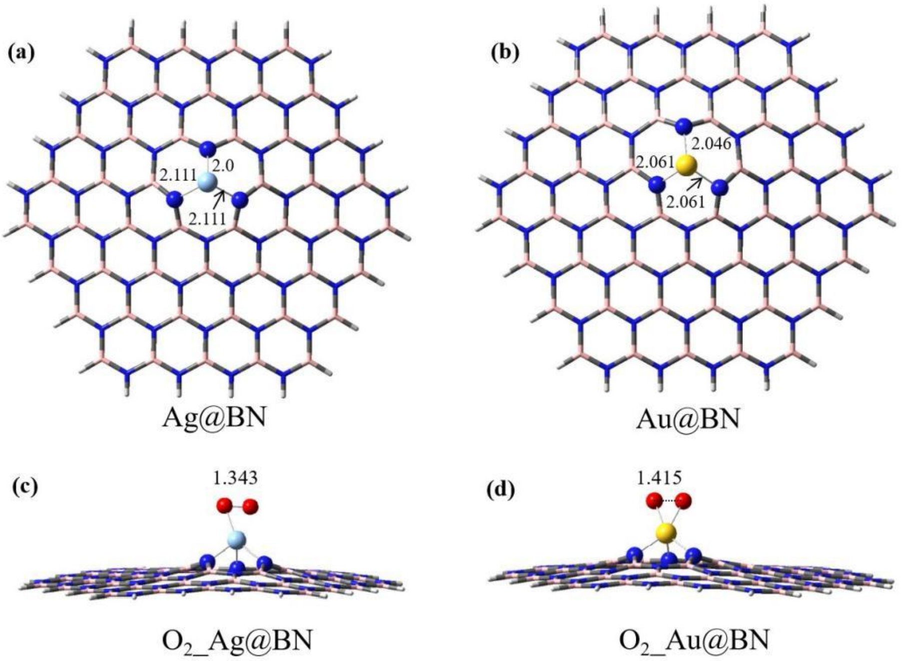

Fig. S1. The larger models for (a)Ag@BN, (b) Au@BN; O_2 adsorption geometries on these SACs: (c) O_2_Ag@BN, (d) O_2_Au@BN.

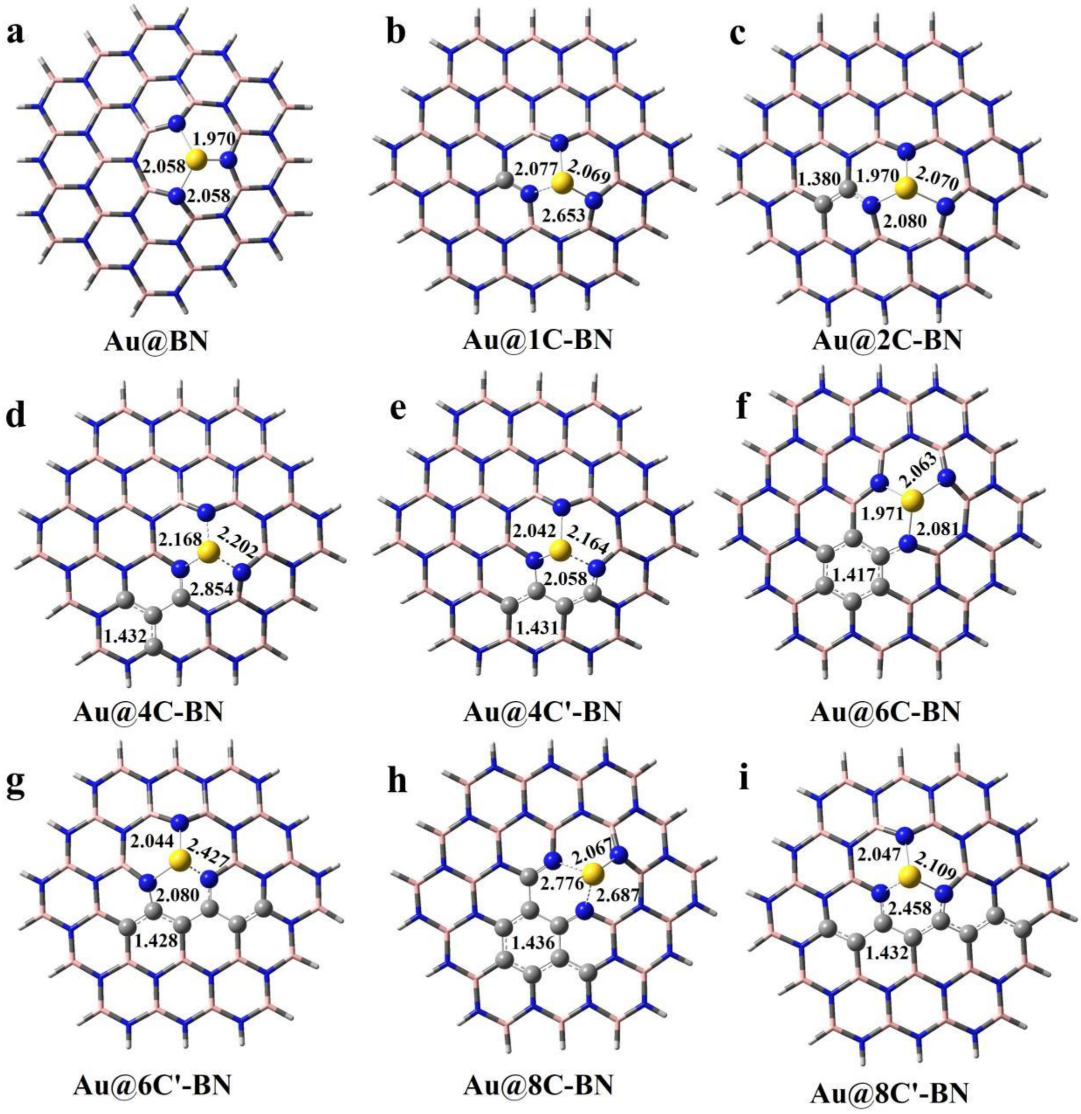

Fig. S2. The optimized geometries of Au anchored at the V_B site of BN or BNC supports.

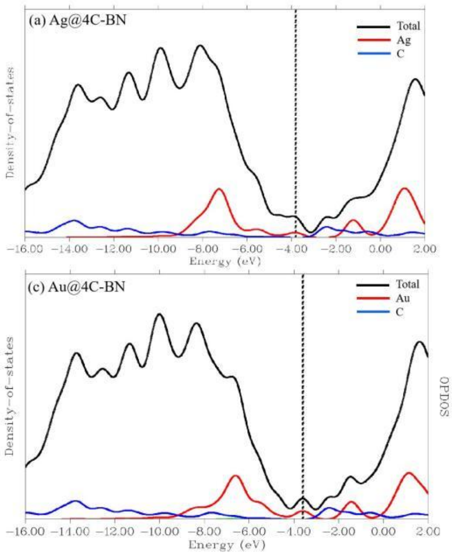

Fig. S3. The total density of states (DOS) and partial density of states (PDOS) of (a) Ag@4C-BN, (b) Ag@6C-BN, (c)Au@4C-BN, and (d) Au@6C-BN.

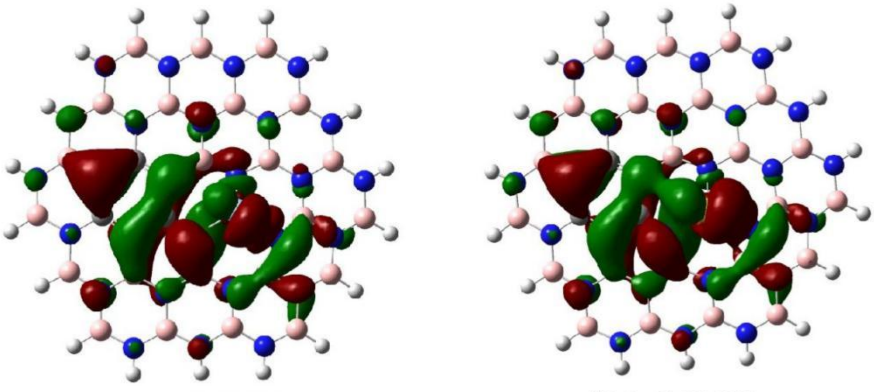

(a) Ag@6C-BN (b) Au@6C-BN

Fig. S4 The HOMO diagrams of (a) Ag@6C-BN and (b) Au@6C-BN (isosurface value = 0.02 a.u.).

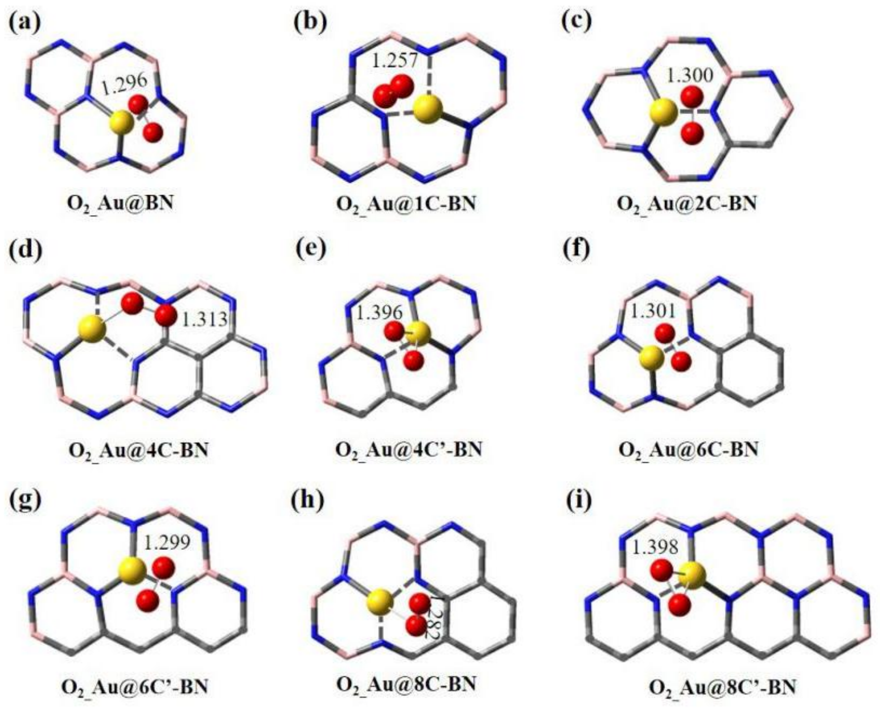

Fig. S5. Optimized adsorption configurations of triplet O_2 on Au@BN and Au@nC-BN, only section of h-BN is displayed.

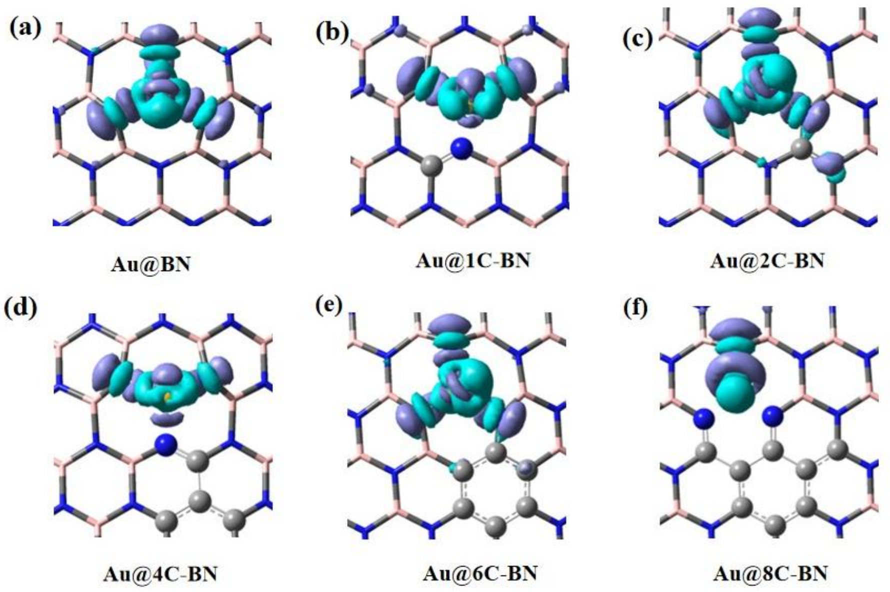

Fig. S6. Electron density difference (EDD) plots for Au@BN and Au@nC-BN systems (Isosurface value= 0.02 a.u.).

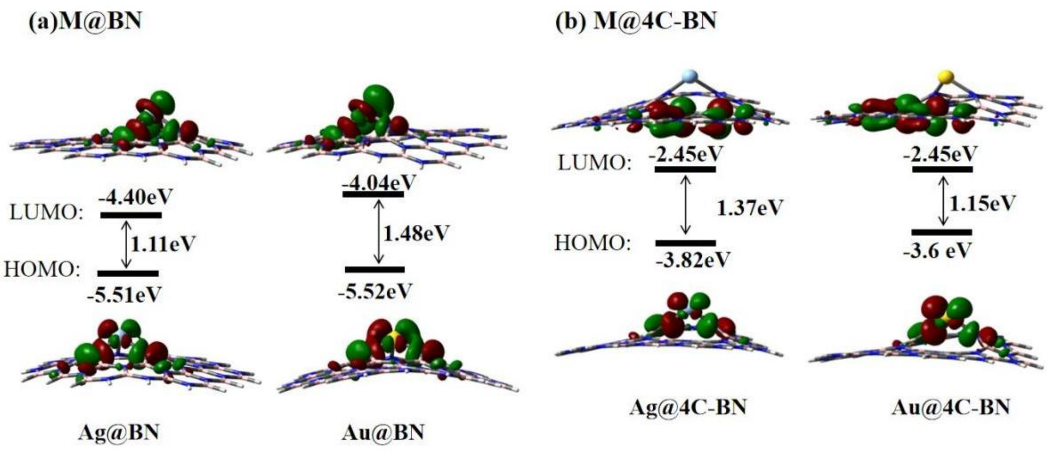

Fig. S7. HOMO and LUMO energy level diagrams for M@BN and M@4C-BN systems.

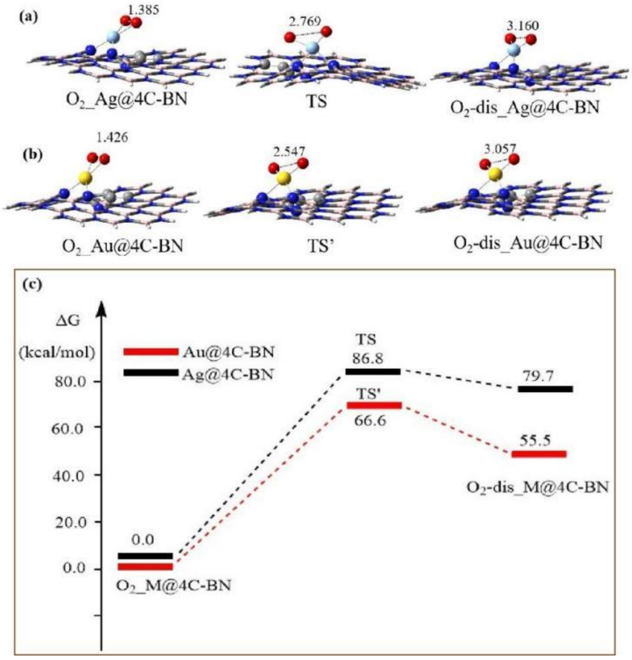

Fig. S8. Optimized geometries of O_2 dissociation on (a) Ag@4C-BN, (b) Au@4C-BN, and (c) the energy profile along O_2 dissociation process.

Table S1. Binding Energy (E_b) of M@BN(M= Ag, Au) and adsorption energy (E_{ads}) of O_2 on original and larger Models (unit: kcal/mol)

Models	SACs	E_b	E_{ads}
$B_{27}N_{27}H_{18}$	Ag@BN	−61.3	7.0
	Au@BN	−62.9	−6.4
$B_{48}N_{48}H_{24}$	Ag@BN	−61.6	6.2
	Au@BN	−63.7	−6.9

Table S2. Adsorption energies (E_{ads}, kcal/mol), adsorption Gibbs free energies (G_{ads}, kcal/mol) for triplet O_2 on Ag and Au single-atom catalysts.

Models	M@BN (a)	M@1C-BN (b)	M@2C-BN (c)	M@4C-BN (d)	M@4C'-BN (e)	M@6C-BN (f)	M@6C'-BN (g)	M@8C-BN (h)	M@8C'-BN (i)
$E_{ads, Ag}$	−22.2	−7.6	−27.2	−20.4	−19.2	−20.5	−21.3	−13.9	−22.1
$E_{ads, Au}$	−26.4	−8.0	−31.3	−30.3	−22.3	−27.1	−31.0	−20.6	−24.4
$G_{ads, Ag}$	−11.2	2.4	−16.3	−8.0	−6.7	−9.1	−10.0	−2.4	−11.8
$G_{ads, Au}$	−13.1	2.9	−17.6	−17.1	−8.9	−13.7	−17.0	−7.2	−10.6

Table S3. Adsorption energies (E_{ads}, kcal/mol), adsorption Gibbs free energies (G_{ads}, kcal/mol) and charge analysis for singlet O_2 adsorption on Ag single-atom catalysts.

Models	Ag@BN (a)	Ag@1C-BN (b)	Ag@2C-BN (c)	Ag@4C-BN (d)	Ag@4C'-BN (e)	Ag@6C-BN (f)	Ag@6C'-BN (g)	Ag@8C-BN (h)	Ag@8C'-BN (i)
E_{ads}	7.0	−10.5	4.4	−17.0	3.3	1.3	−0.1	−4.5	1.9
G_{ads}	20.7	3.4	18.2	−1.6	17.7	16.6	15.7	11.3	16.8
$Q(O_2)$	−0.46	−0.28	−0.45	−0.73	−0.54	−0.53	−0.57	−0.61	−0.56
$\Delta Q(M)$	0.08	0.09	0.08	0.27	0.09	0.06	0.10	0.23	0.12

Table S4. Adsorption energies (E_{ads}, kcal/mol), adsorption Gibbs free energies (G_{ads}, kcal/mol) and charge analysis for singlet O_2 adsorption on Au single-atom catalysts.

Models	Au@BN (a)	Au@1C-BN (b)	Au@2C-BN (c)	Au@4C-BN (d)	Au@4C'-BN (e)	Au@6C-BN (f)	Au@6C'-BN (g)	Au@8C-BN (h)	Au@8C'-BN (i)
E_{ads}	−6.4	−20.8	−2.4	−33.4	−9.8	−9.8	−13.6	−20.3	−13.0
G_{ads}	8.4	−6.3	12.6	−18.0	4.7	5.5	1.8	−3.5	2.0
$Q(O_2)$	−0.59	−0.32	−0.54	−0.78	−0.60	−0.60	−0.60	−0.70	−0.60
$\Delta Q(M)$	0.20	0.18	0.13	0.41	0.12	0.10	0.13	0.44	0.15
